# Pulmonary metastasectomy in colorectal cancer: health utility scores by EQ-5D-3L in a randomized controlled trial show no benefit from lung metastasectomy

**DOI:** 10.1111/codi.15386

**Published:** 2020-10-21

**Authors:** Chris Brew-Graves, Vernon Farewell, Kathryn Monson, Mišel Milošević, Norman R. Williams, Eva Morris, Fergus Macbeth, Tom Treasure, Lesley Fallowfield

**Affiliations:** 1 National Cancer Imaging Translational Accelerator (NCITA), Division of Medicine, UCL, London, UK; 2 MRC Biostatistics Unit, University of Cambridge, Cambridge, UK; 3 Sussex Health Outcomes Research and Education in Cancer (SHORE-C), Brighton and Sussex Medical School, University of Sussex, Falmer, UK; 4 Thoracic Surgery Clinic, Institute for Lung Diseases of Vojvodina, Sremska Kamenica, Serbia; 5 Surgical and Interventional Trials Unit (SITU), University College London, London, UK; 6 Nuffield Department of Population Health, Big Data Institute, University of Oxford, Oxford, UK; 7 Centre for Trials Research, Cardiff University, Cardiff, UK; 8 Clinical Operational Research Unit, University College London, London, UK

**Keywords:** lung metastasectomy, colorectal cancer, randomized controlled trial

## Abstract

**Aim:**

The aim was to assess the health utility of lung metastasectomy in the treatment of patients with colorectal cancer (CRC) using the EQ-5D-3L questionnaire.

**Methods:**

Multidisciplinary CRC teams at 14 sites recruited patients to a two-arm randomized controlled trial—Pulmonary Metastasectomy in Colorectal Cancer (PulMiCC). Remote randomization was used, stratified by site and with minimization for seven known confounders. Participants completed the EQ-5D-3L questionnaire together with other patient reported outcome measures at randomization and then again at 3, 6, 12 and 24 months. These were returned by post to the coordinating centre.

**Results:**

Between December 2010 and December 2016, 93 participants were randomized, 91 of whom returned questionnaires. Survival and patient reported quality of life have been published previously, revealing no significant differences between the trial arms. Described here are patient reported data from the five dimensions of the EQ-5D-3L and the visual analogue scale (VAS) health state. No significant difference was seen at any time point. The estimated difference between control and metastasectomy patients was −0.23 (95% CI –0.113, 0.066) for the composite 0 to 1 index scale based on the descriptive system and 0.123 (95% CI –7.24, 7.49) for the 0 to 100 VAS scale.

**Conclusions:**

Following lung metastasectomy for CRC, no benefit was demonstrated for health utility, which alongside a lack of a survival or quality of life benefit calls into question the widespread use of the procedure.

## 1 Introduction

The results of the Pulmonary Metastasectomy in Colorectal Cancer (PulMiCC) randomized controlled trial (RCT) showed no survival benefit from lung metastasectomy for colorectal cancer (CRC). Hence any survival benefit that might be revealed by a much larger trial is likely to be far smaller than has generally been supposed [1]. Quality of life (QoL) in PulMiCC included four patient reported outcome measures: the general and anaemia scale of the Functional Assessment of Cancer Therapy (FACT-G-An) [2], selected items from the lung cancer brief symptom index [3] and the short form of the Spielberger State/Trait Anxiety Inventory (STAI) [4]. These were chosen as the most relevant assessments for a thoracic oncological surgical intervention. No significant differences were found between the control and metastasectomy arms of the trial for any QoL outcomes and minimally important differences in these measures were largely ruled out [5].

**Figure 1:**
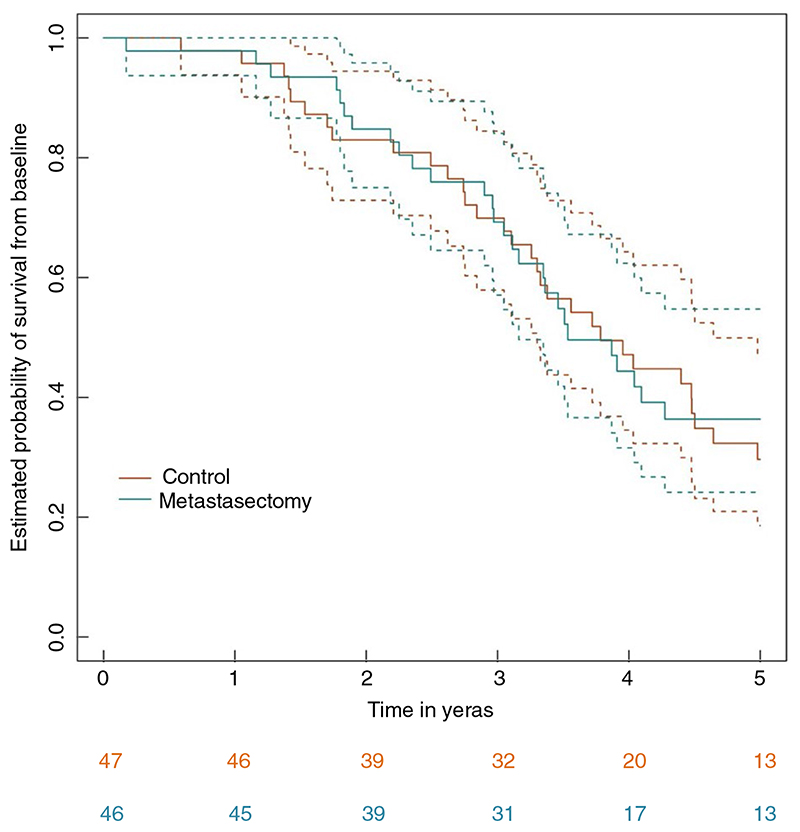
Survival in the PulMiCC trial to 5 years

In the absence of a demonstrable benefit in either survival or QoL in PulMiCC, we examined the health utility of surgical removal of CRC lung metastases. Here we report analysis of the data from the EQ-5D-3L questionnaire. This is a standardized health utility questionnaire, developed by the EuroQol Group that provides a simple, generic measure of health for clinical and economic appraisal [6]. It is most often used in health economics studies to generate quality adjusted life years. The PulMiCC EQ-5D-3L results reported here complement the trial survival data [1] (Figure 1) and will contribute to a future health economics evaluation.

## 2 Methods

As full details of the RCT have been previously reported [1,5] the trial design is provided only briefly. PulMiCC had two stages of consent. First, following written informed consent, patients with lung metastases were registered for assessment. Those subsequently found to be eligible for metastasectomy, according to current practice, were invited to consent to randomization, which was either to continued standard care (control) or metastasectomy. Sussex Health Outcomes Research and Education in Cancer (SHORE-C), University of Sussex, administered and coordinated all patient reported outcome measures.

The full trial protocol can be accessed online: https://www.ucl.ac.uk/clinical-operational-research-unit/sites/clinical-operational-research-unit/files/pulmicc_protocol_december_2015.pdf. In arm 1, Control, patients were managed without metastasectomy, radiotherapy or image guided thermal ablation. In arm 2, Pulmonary Metastasectomy, the surgical approach (videothoracoscopy or open thoracotomy) was at the discretion of the surgeon.

### 2.1 Participants

Patients were eligible for inclusion if they had undergone resection of primary CRC with a prospect of cure and had pulmonary metastasis confirmed at a multidisciplinary team meeting. The discovery of the metastases could be synchronous or metachronous. In line with usual practice, there had to be no clinical indications of active CRC other than the known lung metastases. Prior liver resection for metastases did not preclude entry to the trial.

Following consent to randomization, patients were given the EQ-5D-3L questionnaire [7] at baseline. Subsequently, it was administered at 3, 6, 12 and 24 months. The questionnaire asks patients to indicate on a visual analogue scale (VAS) their own health state ‘today’, between zero, worst imaginable health state, and 100, best imaginable health state, and to indicate their well-being in five dimensions (mobility, self-care, usual activity, pain and discomfort, anxiety and depression) as 1, 2 or 3, the three levels denoting no, some and extreme problems respectively. Country-specific composite indices, on a scale of 0 (worst) to 1 (best), based on the five well-being scores were developed. No index was found for Serbia so the UK index was calculated for all patients where the majority of trial centres were based. Of the randomized patients, 70% were in the UK, and Serbian patients were similarly represented in both arms by stratification.

### 2.2 Statistical methods

To analyse the longitudinal EQ-5D-3L health state and index data, with adjustment for within-patient correlation, we used linear regression models with estimation using generalized estimating equations, using an independence working covariance assumption. The primary analysis estimated a common effect of metastasectomy over the follow-up times of 3, 6, 12 and 24 months, with adjustment for follow-up time, but variation of the treatment effect over time was examined. The potential impact of losses to follow-up was examined through fitting singular linear increment models [8].

## 3 Results

Fourteen sites randomized 93 patients (Table 1) 47 to the control arm and 46 to metastasectomy. No patient in the control group had a metastasectomy as their initial treatment; two had metastasectomy later at 14 and 17 months. Two patients declined the assigned metastasectomy. For this analysis, they remain in their assigned groups.

Of 93 randomized patients, one in each arm did not complete any EQ-5D-3L questionnaires, leaving 46 control and 45 metastasectomy patients. Fourteen patients died within 24 months: 8/46 of controls and 6/45 who had metastasectomy (Table 2).

**Figure 2:**
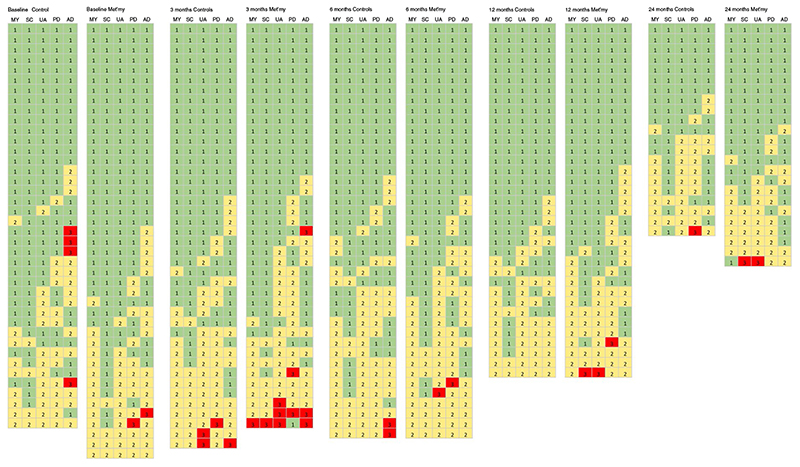
Three-level (3, 2, 1) scores in a traffic light convention, in the five dimensions of well-being in the EuroQol: mobility, selfcare, usual activity, pain and discomfort, anxiety and depression (EQ-5D-3L) at baseline, 3, 6, 12 and 24 months, in the control (left) and metastasectomy arms. Each horizontal set of five represents a return from an individual patient. At each time point they are ranked by the unadjusted sum of the scores from 5 at the top, to help visibility of the patterns between the arms and over time

Summary tabulations of the five EQ-5D-3L well-being components are provided in Figure 2. The three levels 1, 2 and 3 are colour coded with a traffic light convention, for each of the five dimensions, for every patient returning a form. The green ‘no problems’ area diminished at a similar rate in controls and metastasectomy patients.

**Figure 3:**
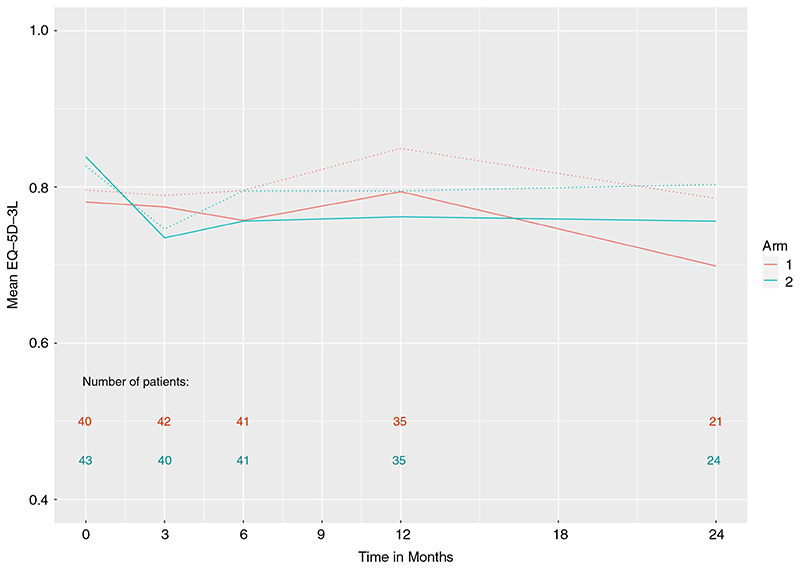
EQ-5D-3L index scores in the two treatment arms: arm 1, control; arm 2, assigned to metastasectomy. Dashed lines are based on generalized estimating equations and solid lines on singular linear models that adjust for drop-out

Figure 3 presents the EQ-5D-3L index values, derived from the well-being components, over the 24 months of follow-up. The estimated effect, comparing metastasectomy with control, was −0.023, 95% CI −0.113, 0.066, *P* = 0.57. There was no evidence that the treatment difference varied over time (*P* = 0.87, three d.f. test). Reported minimally important differences for this measure in a UK population range from 0.10 to 0.12, suggesting that there is no evidence of any important difference in the index values between the randomized groups [9].

**Figure 4:**
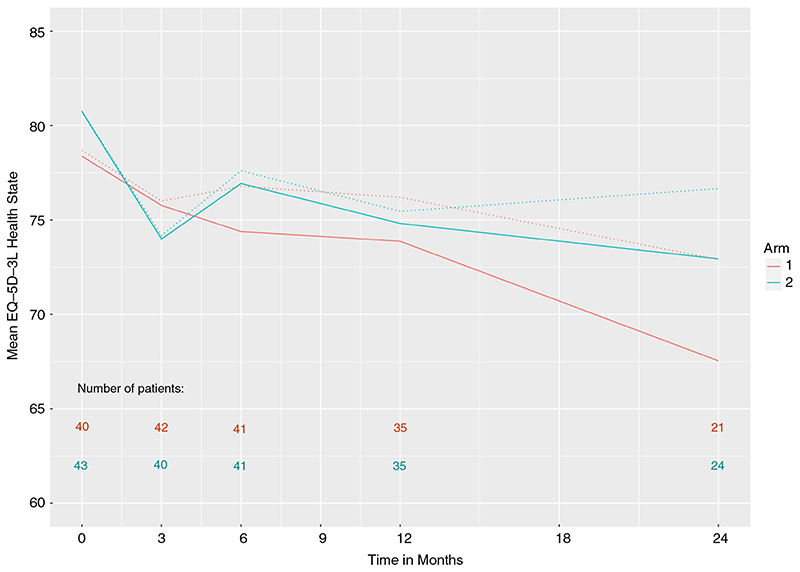
EQ-5D-3L health state scores in the two treatment arms: arm 1, control; arm 2, assigned to metastasectomy. Dashed lines are based on generalized estimating equations and solid lines on singular linear models that adjust for drop-out

Figure 4 presents the EQ-5D-3L health state scores over the 24 months of follow-up. The estimated effect, comparing metastasectomy with control, was 0.125, 95% CI −7.24, 7.49, *P* = 0.97. There was no evidence that the treatment difference varied over time (*P* = 0.87, three d.f. test).

**Figure 5:**
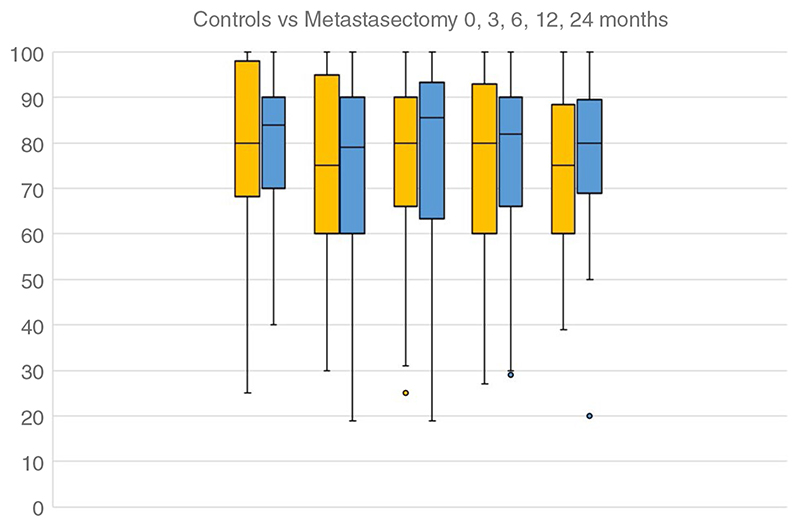
The single dimension, self-reported health state on a 1-100 scale with median and interquartile range displayed

Figure 5 presents the single dimension, self-reported health state on a 1–100 scale with median and interquartile range. It fell in both groups at similar rates.

## 4 Discussion

Data reported here from the EQ-5D-3L well-being dimensions and health state show no differences between the randomized control and metastasectomy patients. This outcome is in line with the finding of no survival or QoL benefit in the PulMiCC RCT [1,5]. Lung metastasectomy is sometimes considered for psychological benefit but, as previously shown when using a comprehensive assessment of anxiety (STAI), no difference was evident in the anxiety and depression dimension of the EQ-5D-3L. The number of patients reporting ‘no problems’ in all the five dimensions of well-being diminished at a similar rate in controls (left) (Figure 2) and metastasectomy patients.

Evaluation of treatment of metastatic disease is a research priority for the Association of Coloproctology of Great Britain and Ireland (ACPGBI) [10] and is one of the targets in management of patients with CRC. It is a treatment considered in the Improving Management of Patients with Advanced Colorectal Tumours, the IMPACT initiative of the ACPGBI [11]. Small effects cannot be ruled out by the findings of PulMiCC but they do not show a survival or QoL benefit from metastasectomy [5]. The additional study results reported here make it unlikely that there is a significant gain of health utility if patients are subjected to pulmonary metastasectomy. PulMiCC trial results may help to guide further research in this important area.

## Figures and Tables

**Table 1:** Trial sites and number of patients returning any forms

Site	Returns
Serbia, Institute for Lung Diseases of Vojvodina	28
Sheffield, Northern General Hospital	16
Basildon, Basildon Hospital	8
Middlesbrough, James Cook Hospital	7
Liverpool, Heart and Chest Hospital	7
Burton, Queen's Hospital	6
Bristol, Royal Infirmary	5
Manchester, Christie Hospital	4
London, Royal Free Hospital	3
Plymouth, Derriford Hospital	2
Wolverhampton, New Cross Hospital	2
London, Royal Brompton Hospital	2
Leicester, Glenfield Hospital	1

**Table 2:** Forms were sent at 3, 6, 12 and 24 months

Time point (months)	0	3	6	12	24
Data available (*n* = 91 patients^a^)	83	84	82	72	60
Per cent return	91	92	90	79	66

*Note:* We know from survival data that, by 24 months, the number of deaths was 8/46 control, 6/45 metastasectomy.

a Ninety-three patients randomized. EQ-5D-3L questionnaire data available for *n* = 91 patients.

## Data Availability

All data are available by an approach to the Chief Investigator and the Trial Centre (SITU, UCL).

## What does this paper add to the literature?

Surgical excision of lung metastases from colorectal cancer provides no health utility benefit among 91 patients in the PulMiCC randomized controlled trial.
